# Independent and joint influence of depression and advanced lung cancer inflammation index on mortality among individuals with chronic kidney disease

**DOI:** 10.3389/fnut.2024.1453062

**Published:** 2024-10-23

**Authors:** Jie Zhou, Wenjun Liu, Xiaoxin Liu, Jijun Wu, Ying Chen

**Affiliations:** ^1^NHC Key Laboratory of Hormones and Development, Tianjin Key Laboratory of Metabolic Diseases, Chu Hsien-I Memorial Hospital and Tianjin Institute of Endocrinology, Tianjin Medical University, Tianjin, China; ^2^Department of Gastrointestinal Oncology Surgery, Hubei Cancer Hospital, Tongji Medical College, Huazhong University of Science and Technology, Wuhan, China; ^3^Department of Vascular Surgery, Guangdong Provincial Key Laboratory of Major Obstetric Diseases, Guangdong Provincial Clinical Research Center for Obstetrics and Gynecology, The Third Affiliated Hospital, Guangzhou Medical University, Guangzhou, China; ^4^Department of Nephrology, Liyuan Hospital, Tongji Medical College, Huazhong University of Science and Technology, Wuhan, China; ^5^Department of Interventional Radiology, Zhongshan Torch Development Zone People's Hospital, Zhongshan, China; ^6^Third Clinical School, Guangzhou Medical University, Guangzhou, China

**Keywords:** inflammatory, nutritional, chronic kidney disease, depression, mortality, NHANES

## Abstract

**Background:**

The combined effect of depression and nutritional-inflammatory status on mortality in the chronic kidney disease (CKD) population is unclear.

**Methods:**

We prospectively analyzed 3,934 (weighted population: 22,611,423) CKD participants from the National Health and Nutrition Examination Survey (2007–2018). Depression and nutritional-inflammatory status were assessed with Patient Health Questionnaire 9 (PHQ-9) and Advanced Lung Cancer Inflammation Index (ALI), respectively. Weighted multivariate COX regression models, restricted cubic splines (RCS) models, and stratified analyses were used to investigate the association of PHQ-9 scores and ALI with all-cause mortality.

**Results:**

During a median follow-up of 5.8 years (interquartile range 3.4–8.6 years), a total of 985 patients died (25.0%). Each point increase in a patient’s PHQ-9 score increased the risk of all-cause mortality by 4% (HR, 1.04; 95% CI, 1.02–1.06; *p* < 0.001), in the full adjusted model. However, an increase in ALI levels was associated with a decreased risk. HRs (95% CI) of 0.76 (0.65–0.90), 0.70 (0.57–0.86), and 0.51 (0.41–0.64) in the Q2, Q3, and Q4 of ALI compared with the Q1 of ALI, respectively. In addition, the joint analysis showed that CKD patients without depression and with higher ALI were associated with a reduced risk of all-cause mortality. Namely, patients in the highest ALI group (Q4) without depression had the lowest risk (HR, 0.32; 95% CI, 0.21–0.48). Furthermore, this combined effect was consistent across all subgroups, and no significant interaction was found (*p* > 0.05 for interaction).

**Conclusion:**

In a nationally representative sample of US patients with CKD, coexisting depression and poorer nutrition-inflammation were associated with a significantly increased risk of all-cause mortality.

## 1 Introduction

Chronic kidney disease (CKD) is a worldwide health problem that is widespread and serious, directly leading to the global burden of incidence and mortality and is also an essential risk factor for cardiovascular disease (CVD) (1, 2). The prevalence of CKD among adults in the US was about 15 percent in 2015–2018, and that number appears to be increasing (3, 4). Notably between 1990 and 2017, the global all-age mortality rate for CKD increased by 41.5% (2). The long-term prognosis of patients with CKD is related to a variety of factors, including nutritional status, degree of inflammation, and psychological factors (5–7). Interestingly, they are interrelated and interact with each other (8–10). Therefore, identifying these modifiable prognostic factors is urgent for the survival of CKD patients.

Recently, multiple nutritional/inflammatory indicators have been increasingly shown to serve as valid predictors of prognosis in chronic kidney disease (6, 11–13). The advanced lung cancer inflammation index (ALI) is a new indicator of systemic inflammation level and nutritional status, which consists of neutrophil to lymphocyte ratio (NLR), serum albumin, and body mass index (BMI), and it showed superior predictive prognostic ability for cancer patients relative to other metrics (14–16). Notably, the predictive properties of ALI for prognosis in CKD patients have not been explored.

In addition, psychological factors also have a greater impact on the prognosis of CKD. Depression is among the most widespread psychological disturbances in patients with CKD, affecting an estimated one in four patients (17, 18). Interestingly, there is a bidirectional association between depression and CKD, which may lead to the disease occurring in conjunction with each other (19). Depressed CKD patients tend to have worse outcomes (20, 21). The relationship between depressive symptoms and nutrient-inflammatory states is close and complex (8, 22–25). The causal relationship between them is controversial, and some studies have suggested a bidirectional link (8, 22, 25–27). A recent meta-analysis of cross-sectional associations found significantly higher levels of pro-inflammatory biomarkers, C-reactive protein, interleukin 6 (IL-6), and tumor necrosis factor-alpha, and significantly lower levels of the anti-inflammatory cytokine IL-10 in CKD and end-stage renal failure patients with depressive symptoms relative to CKD and end-stage renal failure patients without depressive symptoms (28). Although many studies explored the relationship between nutritional-inflammatory status and depression, no studies have examined the significance of the combination of nutritional-inflammatory status and depression on the prognosis of CKD patients.

We conducted a prospective study through a nationally representative sample of CKD in the US. First, to examine the performance of ALI for mortality prediction and compare it with other indicators of nutritional/inflammation (based on blood counts). Subsequently, it was used to represent the nutritional-inflammatory status, and we then explored the independent and joint effects of this indicator and depression on the risk of all-cause mortality.

## 2 Methods

### 2.1 Study population

This prospective cohort study used data from a nationally representative sample of six consecutive cycles of the National Health and Nutrition Examination Survey (NHANES) from 2007 to 2018. The Ethics Review Board of the National Center for Health Statistics approved all NHANES protocols, and written informed consent was obtained from all participants. The survey has been conducted every 2 years since 1999 to monitor the health and nutritional status of the US population through interviews and physical examinations.

NHANES uses stratified, multistage random sampling to collect data from a nationally representative, noninstitutionalized US sample. Participants were considered to have CKD when they presented with an estimated glomerular filtration rate (eGFR) < 60 mL /min/1.73 m^2^ or urinary albumin/creatinine ratio (UACR) ≥ 30 mg/g (29). The eGFR was calculated by the CKD-EPI equation (30). Among the 59,482 participants from 2007 to 2018, we excluded those (a) with incomplete Patient Health Questionnaire-9 (PHQ-9) (*n* = 28,022); (b) who were pregnant (*n* = 323); (c) less than 20 years old (*n* = 1,616); (d) without CKD (*n* = 24,222); (e) with missing mortality data (*n* = 6); (f) with missing data on nutritional/inflammatory indicators (*n* = 342); (g) any missing covariate information (*n* = 1,017). A sample of 3,934 (weighted population: 22,611,423) eligible CKD patients were finally included in this study.

### 2.2 Definition of depression

We used PHQ-9, which includes nine items (lack of interest, depressed mood, trouble sleeping, fatigue, appetite problems, worthlessness, lack of concentration, psychomotor agitation or retardation, and suicidal thoughts), to determine the depression in the CKD population. The score for each item is based on how often the participant has experienced a particular symptom in the past 2 weeks. Each project has four answer categories, “not at all,” “several days,” “more than half the days,” and “nearly every day,” corresponding to 0, 1, 2, and 3 points, respectively. As a result, the total score on the PHQ-9 ranges from 0 to 27, with higher scores representing more severe depressive symptoms. Based on extensive research on the accuracy of the PHQ-9, we defined participants with a total score ≥ 10 as having depression (31, 32).

### 2.3 Assessment of nutritional/inflammatory indicators

ALI was obtained by multiplying BMI (kg/m^2^) by serum albumin (g/dL) divided by NLR. A higher ALI represents better nutritional status and lower levels of inflammation in the participants. Detailed calculations of other nutritional/inflammatory indicators were shown in Supplementary Table S1.

### 2.4 Assessment of mortality

The study endpoint for this cohort was all-cause mortality, meaning death due to any cause. Participants’ vital status and the length of follow-up were determined by cross-referencing with the National Death Index until December 31, 2019.^1^ The follow-up period was counted from initial participation in the NHANES program to the date of death or December 31, 2019.

### 2.5 Covariates

The covariates in this study consisted mainly of demographic characteristics (age, sex, race, education level, income-poverty ratio [PIR], and marital status), medical history information (diabetes mellitus [DM], CVD, hyperlipidemia, hypertension, cancer, smoking status, drinking status, and sleep duration) and laboratory blood indicators (high-density lipoprotein cholesterol [HDL-C], alanine aminotransferase [ALT], aspartate aminotransferase [AST], total cholesterol [TC], and eGFR). Specific groupings of categorical variables and units for all variables can be found in Table 1. Participants were considered to have a history of CVD when they self-reported angina, congestive heart failure, coronary heart disease, heart attack, and stroke. When participants were asked, “Have you ever been told by a doctor or other health professional that you have cancer or any malignant tumor?,” patients were considered to have a history of cancer if they answered yes. All other variable definitions are available in this study (33). Exhaustive measurement techniques for all variables in this study are available at https://www.cdc.gov/nchs/nhanes/.

**Table 1:** Baseline characteristics of chronic kidney disease participants stratified by quartiles of advanced lung cancer inflammation index.

Characteristic	Overall	Advanced lung cancer inflammation index				*p* value
		Quartile 1 (2.8–39.7)	Quartile 2 (39.7–56.9)	Quartile 3 (56.9–80.2)	Quartile 4 (80.2–17930.1)	
Age (years), mean (SE)		65.2 (0.5)	60.5 (0.6)	58.6 (0.5)	57.3 (0.5)	<0.001
Sex, *n* (weighted %)						0.037
Female	2,033 (56.3)	430 (52.2)	512 (57.1)	531 (55.0)	560 (61.1)	
Male	1,901 (43.7)	554 (47.8)	471 (42.9)	452 (45.0)	424 (38.9)	
Race, *n* (weighted %)						<0.001
Mexican American	506 (7.0)	103 (5.0)	139 (8.0)	150 (7.1)	114 (7.8)	
Non-Hispanic Black	876 (11.6)	140 (6.8)	173 (8.9)	205 (10.7)	358 (20.6)	
Non-Hispanic White	1,885 (70.7)	593 (78.5)	502 (73.0)	455 (71.2)	335 (59.5)	
Other Hispanic	330 (4.4)	67 (3.3)	85 (4.6)	83 (4.1)	95 (5.6)	
Other	337 (6.3)	81 (6.3)	84 (5.4)	90 (6.9)	82 (6.4)	
Marital status, *n* (weighted %)						0.284
Married/living with partner	2,165 (59.7)	522 (56.5)	559 (61.1)	564 (61.3)	520 (59.5)	
Divorced, separated, widowed, or never married	1,769 (40.3)	462 (43.5)	424 (38.9)	419 (38.7)	464 (40.5)	
Poverty income ratio, *n* (weighted %)						0.014
<1.3	1,366 (24.8)	320 (22.9)	344 (26.6)	344 (23.1)	358 (26.8)	
1.3 to <3.5	1,630 (40.4)	437 (45.5)	411 (40.5)	378 (36.4)	404 (39.6)	
≥3.5	938 (34.7)	227 (31.6)	228 (32.9)	261 (40.5)	222 (33.5)	
Education level, *n* (weighted %)						0.435
Less than high school	1,117 (19.5)	281 (20.7)	275 (20.1)	276 (16.6)	285 (20.7)	
High school	971 (25.4)	235 (25.5)	256 (26.7)	238 (24.7)	242 (24.8)	
More than high school	1,846 (55.1)	468 (53.8)	452 (53.2)	469 (58.7)	457 (54.5)	
Smoking, *n* (weighted %)						0.008
Never	1,960 (51.0)	429 (45.4)	490 (50.3)	514 (51.4)	527 (57.0)	
Former	1,289 (33.2)	379 (38.0)	301 (32.2)	311 (34.6)	298 (27.8)	
Now	685 (15.8)	176 (16.6)	192 (17.5)	158 (14.0)	159 (15.2)	
Alcohol use, *n* (weighted %)						0.004
Never	647 (13.7)	155 (13.5)	159 (14.0)	152 (11.8)	181 (15.7)	
Former	965 (20.6)	282 (26.2)	227 (19.4)	233 (17.6)	223 (19.3)	
Mild	1,317 (38.0)	347 (37.6)	343 (39.2)	335 (41.6)	292 (33.0)	
Moderate	469 (14.2)	101 (12.1)	112 (13.9)	123 (16.0)	133 (14.8)	
Heavy	536 (13.5)	99 (10.6)	142 (13.5)	140 (13.0)	155 (17.2)	
Body mass index, *n* (weighted %)						<0.001
<25 kg/m^2^	925 (24.1)	389 (39.5)	259 (26.3)	176 (17.9)	101 (10.3)	
25 to <30 kg/m^2^	1,207 (29.1)	345 (35.1)	320 (32.6)	293 (29.8)	249 (25.3)	
≥30 kg/m^2^	1,802 (46.8)	250 (25.4)	404 (41.1)	514 (52.3)	634 (64.4)	
Sleep duration, *n* (weighted %)						0.028
<7 h	1,318 (29.2)	296 (27.8)	288 (26.6)	355 (30.0)	379 (32.5)	
7–9 h	2,287 (62.6)	587 (61.9)	605 (64.2)	560 (64.5)	535 (59.7)	
>9 h	329 (8.2)	101 (10.3)	90 (9.2)	68 (5.5)	70 (7.8)	
ALT (U/L), mean (SE)	24.7 (0.5)	23.1 (1.4)	24.2 (0.6)	25.2 (0.9)	26.3 (0.6)	0.12
AST (U/L), mean (SE)	26.7 (0.3)	28.1 (1.0)	26.1 (0.5)	26.2 (0.5)	26.6 (0.6)	0.535
HDL-C (mmol/L), mean (SE)	1.4 (0.01)	1.5 (0.02)	1.4 (0.01)	1.3 (0.01)	1.3 (0.01)	<0.001
Total cholesterol (mmol/L), mean (SE)	5.0 (0.02)	4.8 (0.04)	5.0 (0.04)	5.0 (0.04)	5.2 (0.03)	<0.001
eGFR (ml/min/1.73m^2^), mean (SE)	73.0 (0.5)	64.4 (0.9)	73.1 (0.9)	76.6 (0.9)	77.8 (0.9)	<0.001
Hypertension, *n* (weighted %)						0.719
No	1,104 (32.4)	265 (31.0)	297 (34.2)	272 (32.4)	270 (32.0)	
Yes	2,830 (67.6)	719 (69.0)	686 (65.8)	711 (67.6)	714 (68.0)	
Hyperlipidemia, *n* (weighted %)						0.017
No	677 (17.1)	198 (19.8)	158 (15.7)	170 (19.0)	151 (13.8)	
Yes	3,257 (82.9)	786 (80.2)	825 (84.3)	813 (81.0)	833 (86.2)	
Cardiovascular disease, *n* (weighted %)						<0.001
No	2,978 (78.4)	665 (72.0)	746 (76.3)	775 (84.2)	792 (80.8)	
Yes	956 (21.6)	319 (28.0)	237 (23.7)	208 (15.8)	192 (19.2)	
Diabetes mellitus, *n* (weighted %)						0.239
No	1,951 (54.9)	501 (55.5)	499 (55.3)	459 (52.8)	492 (55.9)	
Pre-diabetes mellitus	363 (9.6)	104 (12.0)	89 (9.0)	85 (9.2)	85 (8.1)	
Yes	1,620 (35.5)	379 (32.4)	395 (35.7)	439 (38.0)	407 (36.0)	
Cancer, *n* (weighted %)						<0.001
No	3,286 (81.9)	757 (74.7)	820 (81.9)	844 (84.4)	865 (86.8)	
Yes	648 (18.1)	227 (25.3)	163 (18.1)	139 (15.6)	119 (13.2)	
Depression, *n* (weighted %)						0.298
No (PHQ-9 score < 10)	3,485 (90.4)	877 (8.8)	860 (11.3)	884 (8.2)	864 (10.2)	
Yes (PHQ-9 score ≥ 10)	449 (9.6)	107 (91.2)	123 (88.7)	99 (91.8)	120 (89.8)	

ALT, Alanine aminotransferase; AST, Aspartate aminotransferase; eGFR, estimated glomerular filtration rate; HDL-C, high-density lipoprotein cholesterol.

### 2.6 Statistical analysis

Sample weighting, clustering, and stratification were applied to the analysis in this study due to the complexity of the NHANES sampling design. Baseline characteristics were expressed as quartiles (Q1–Q4) of the ALI. Continuous and categorical variables were, respectively, expressed as weighted means ± standard error (SE) and unweighted frequencies (weighted percentages) and were compared using weighted linear regression and chi-square tests, respectively. We used time-dependent receiver operating characteristic curves (time-ROC) to examine the performance of ALI for mortality prediction and compare it with other indicators of nutritional/inflammation (based on blood counts).

Hazard ratios (HRs) and 95% confidence interval (CI) for the effects of depression and ALI on all-cause mortality were assessed using weighted multivariate Cox proportional hazards models, respectively. Subsequently, we grouped participants in the four ALI levels into eight subgroups based on the presence or absence of depression, to investigate the impact of depression in combination with ALI on the prognosis of patients with CKD. This effect was assessed by weighted multivariate Cox proportional hazards modeling using Q1 ALI participants with depression as controls. A total of three models were constructed. Model 1 was not adjusted. Model 2 was adjusted for demographic characteristics. Model 3 was adjusted for medical history information, and laboratory blood indicators based on Model 2. Harrell et al. concluded that the model fits better when the number of knots is four, i.e., it takes into account the smoothness of the curves while avoiding the reduction in accuracy caused by overfitting (34). Therefore, in this study, the knots of the RCS model are set to four (5th, 35th, 65th, and 95th percentiles) to further explore potential nonlinear associations of PHQ-9 scores and ALI with all-cause mortality.

We repeated the main analyses stratified by sex, age (<65 vs. ≥65 years), PIR (<1.3 vs. 1.3 to <3.5 vs. ≥3.5) and DM (yes vs. pre-DM vs. no). We also used stratification analysis to assess potential interactions. In the stratified analysis of joint influence, due to the small sample size of some groups, we transformed ALI into a binary variable (≤49.98 and >49.98) based on the optimal cut-off of the ROC curve ALI. Subsequently, we performed two sensitivity analyses. In the first analysis, participants who died during the first year of follow-up were excluded from the analysis to reduce the possibility of reverse causality bias. For the second analysis, we unweighted the analyses to compare whether weighted analyses had a large effect on the results.

All statistical analyses were performed using R software version 4.4.0 when a two-sided *p* < 0.05 was considered the threshold for statistical significance.

## 3 Results

### 3.1 Study participants

A total of 3,934 patients with CKD (weighted population: 22,611,423; weighted mean age [SE] 60.4 [0.3] years; weighted female proportion 56.3%). Of these participants, 506 (7.0%) were Mexican American, 1,885 (70.7%) were Non-Hispanic White, 876 (11.6%) were Non-Hispanic Black, 330 (4.4%) were Other Hispanic, and 337 (6.3%) were of Other races. Participants were categorized into 4 groups based on quartiles of the ALI: Q1 (2.8–39.7), Q2 (39.7–56.9), Q3 (56.9–80.2), and Q4 (80.2–17930.1). Their detailed baseline data characteristics were presented in Table 1. Participants with lower ALI were more likely to be older, male, Non-Hispanic White, PIR between 1.3 and 3.5, former alcohol use, former smoking, BMI between 25 and 30 kg/m^2^, sleep duration > 9 h, higher HDL-C, lower total cholesterol, lower eGFR, non-hyperlipidemia, and history of CVD and cancer.

### 3.2 Predictive accuracy of ALI

**Figure 1:**
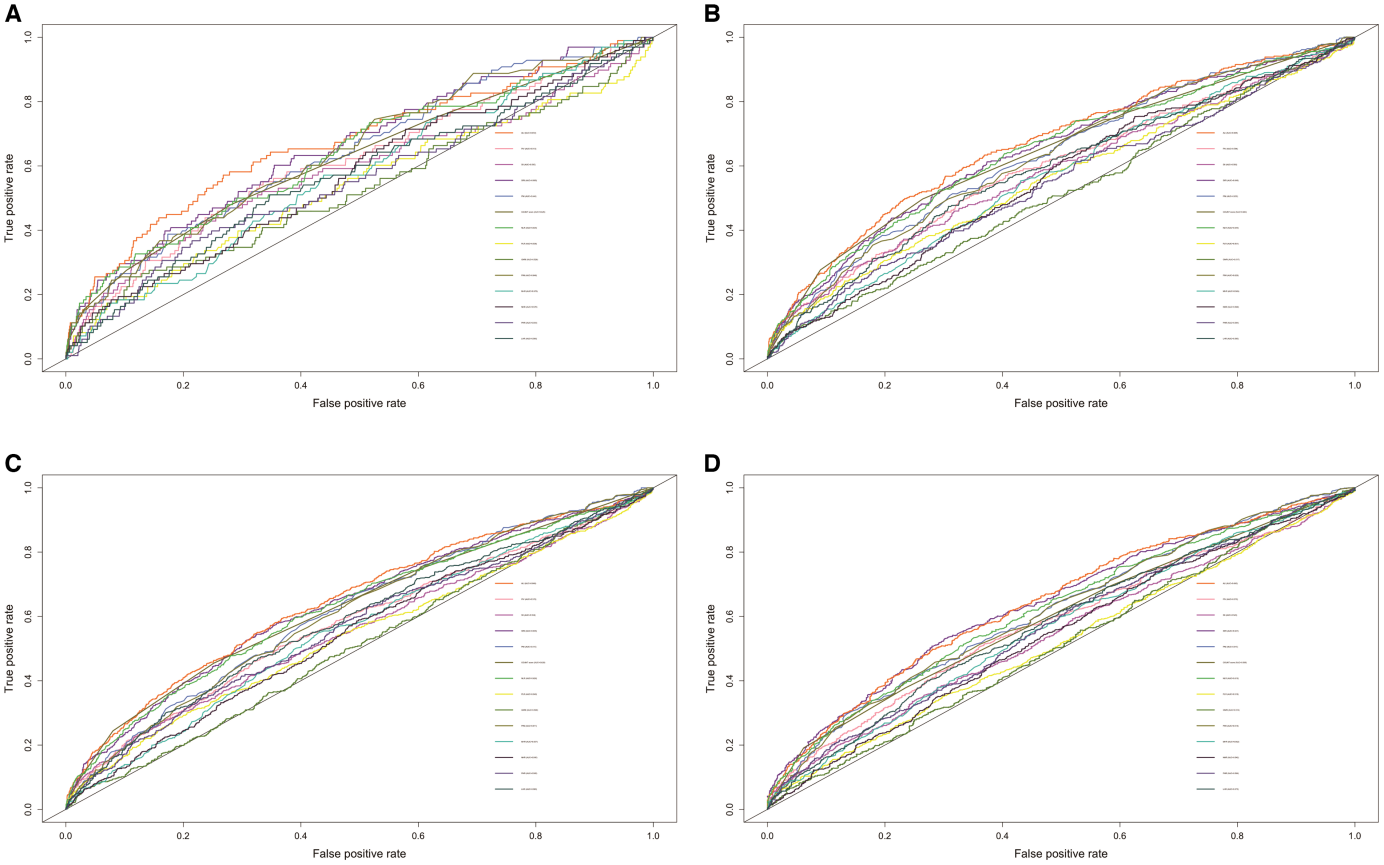
The ROC of nutritional/inflammatory indicators in predicting 1- **(A)**, 3- **(B)**, 5- **(C)**, and 10- **(D)** years all-cause mortality in patients with chronic kidney disease. ROC, receiver operating characteristics curve; AUC, area under the curve; ALI, advanced lung cancer inflammation index; NLR, neutrophil to lymphocyte ratio; SII, systemic immune-inflammation index; SIRI, systemic inflammation response index; PIV, pan-immune-inflammation value; PLR, platelet-to-lymphocyte ratio; PNI, prognostic nutritional index; GNRI, geriatric nutritional risk index; PINI, prognostic immune nutritional index; NHR, neutrophil to high-density lipoprotein-cholesterol ratio; MHR, Monocyte to high-density lipoprotein-cholesterol ratio; PHR, platelet to high-density lipoprotein-cholesterol ratio; LHR, lymphocyte to high-density lipoprotein-cholesterol ratio; COUNT score, controlling nutritional status score.

The effectiveness of 14 nutritional/inflammatory indicators for the prediction of all-cause mortality was assessed using time-ROC. The area under the curve (AUC) for all-cause mortality at 1, 3, 5, and 10 years for ALI was 0.672, 0.669, 0.648, and 0.642, respectively, which was the largest relative to any of the other nutritional/inflammatory indicators (Figure 1).

### 3.3 Independent association of depression and ALI with all-cause mortality

During a median follow-up of 5.8 years (interquartile range 3.4–8.6 years), a total of 985 patients died (25.0%). Weighted multivariate Cox proportional hazards regression model (Model 3) showed that the risk of all-cause mortality was elevated in CKD patients with depression compared to CKD patients without it, with HR of 1.55 (95% CI, 1.24–1.95) (Table 2). In addition, the risk increased by 4% (*p* < 0.001) for each point increase in participants’ PHQ-9 scores. Furthermore, elevated ALI levels were associated with a significantly lower risk (Table 2). Multivariable-adjusted model (Model 3) showed HRs (95% CI) of 0.76 (0.65–0.90), 0.70 (0.57–0.86), and 0.51 (0.41–0.64) in the Q2, Q3, and Q4 of ALI compared with the Q1 of ALI, respectively.

**Table 2:** Association of advanced lung cancer inflammation index (ALI) and PHQ-9 score with all-cause mortality among chronic kidney disease patients.

Subgroup		Hazard ratio (95% CI)		
	No. of deaths (No. of participants)	Model 1^a^	Model 2^b^	Model 3^c^
**PHQ-9 score**				
Per 1 score increase		1.03 (1.01–1.05)	1.05 (1.04–1.07)	1.04 (1.02–1.06)
0–9 (no depression)	857/3,485	1 [Reference]	1 [Reference]	1 [Reference]
10–27 (depression)	128/449	1.41 (1.12–1.77)	1.76 (1.42–2.18)	1.55 (1.24–1.95)
**ALI**				
Quartile 1	390/984	1 [Reference]	1 [Reference]	1 [Reference]
Quartile 2	239/983	0.57 (0.48–0.68)	0.69 (0.58–0.82)	0.76 (0.65–0.90)
Quartile 3	201/983	0.47 (0.39–0.58)	0.64 (0.53–0.79)	0.70 (0.57–0.86)
Quartile 4	155/984	0.35 (0.29–0.42)	0.48 (0.40–0.58)	0.51 (0.41–0.64)
*p* for trend		<0.001	<0.001	<0.001

PHQ-9, Patient Health Questionnaire-9.

a Model 1: No adjustment for covariates.

b Model 2: Adjusted for age, sex, race, PIR, education level, and marital status.

c Model 3: Adjusted for smoking, alcohol use, sleep duration, AST, ALT, HDL-C, total cholesterol, eGFR, hypertension, hyperlipidemia, CVD, DM, and cancer based on Model 2.

**Figure 2:**
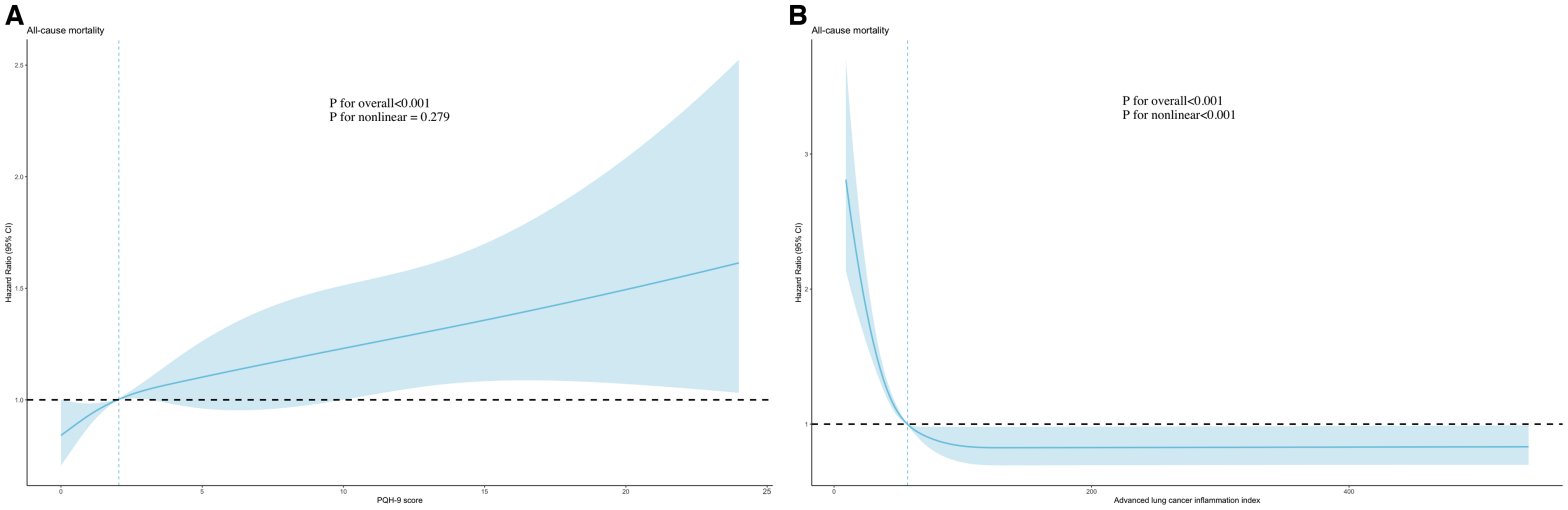
Association of PHQ-9 score **(A)** and advanced lung cancer inflammation index **(B)** with all-cause mortality using RCS models. Adjusted for age, sex, race, PIR, education level, marital status, smoking, alcohol use, sleep duration, AST, ALT, HDL-C, total cholesterol, eGFR, hypertension, hyperlipidemia, CVD, DM, and cancer. PHQ-9, Patient Health Questionnaire-9; RCS, restricted cubic spline.

We observed a nonlinear relationship between PHQ-9 score (*p* for non-linear = 0.019) and ALI (*p* for non-linear<0.001) and the risk of all-cause mortality using the RCS model (adjusted for all covariates) (Figure 2). As the PHQ-9 score increased, the risk first increased sharply and then leveled off. In addition, there was an “L” shaped correlation between the risk and the level of ALI, with the risk initially decreasing significantly with increasing ALI and then leveling off.

### 3.4 Combined association of depression and ALI with all-cause mortality

**Figure 3:**
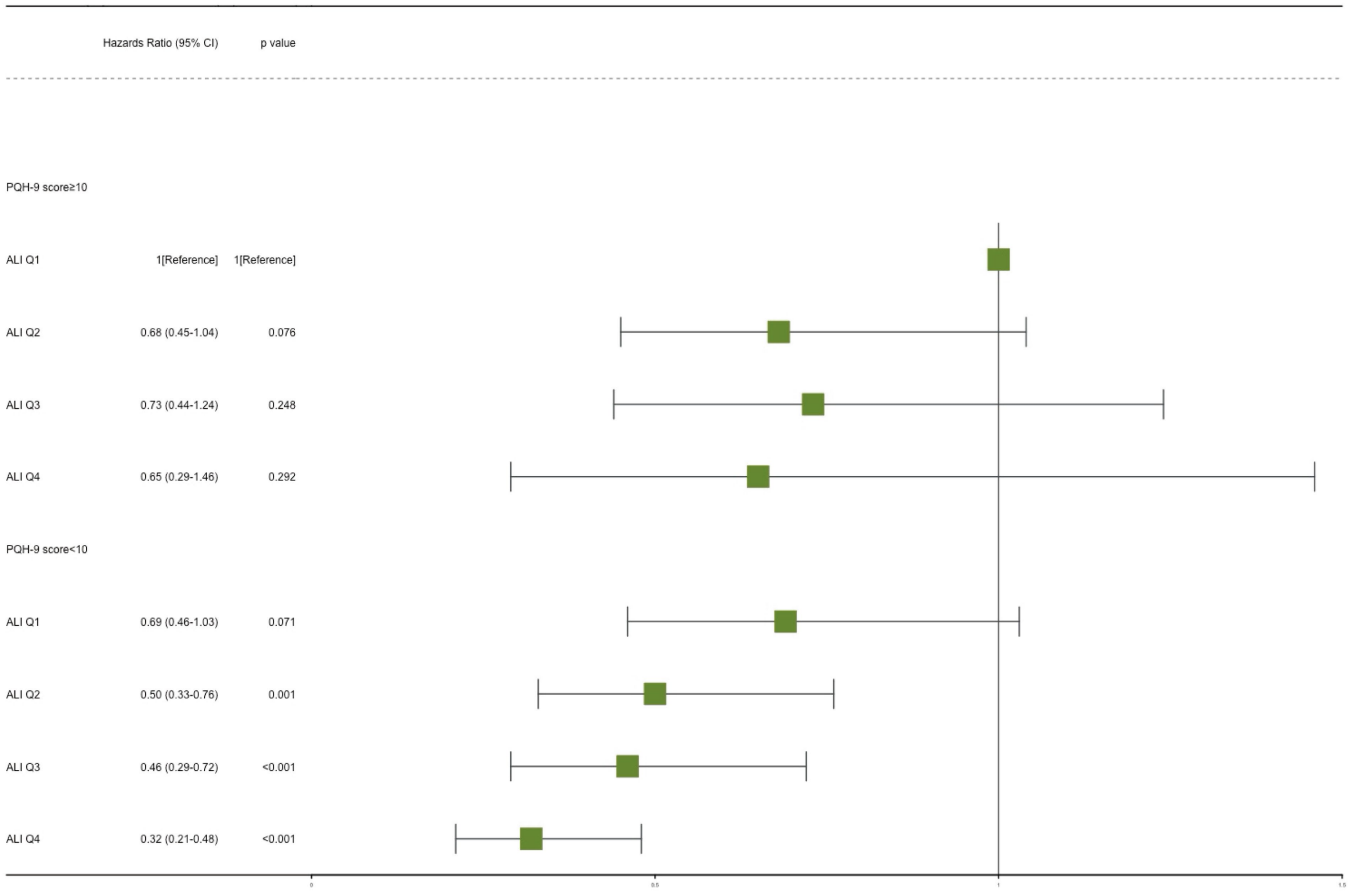
Joint effect analysis of depression and advanced lung cancer inflammation index (ALI) with risks of all-cause mortality. Adjusted for age, sex, race, PIR, education level, marital status, smoking, alcohol use, sleep duration, AST, ALT, HDL-C, total cholesterol, eGFR, hypertension, hyperlipidemia, CVD, DM, and cancer. PHQ-9, Patient Health Questionnaire-9.

The potential joint effect of depression and ALI on all-cause mortality was further explored by combining the presence/absence of the depression group and the ALI group into 8 categories of variables to indicate joint exposure. We observed an increased risk in participants with depression and lower ALI levels (Table 3; Figure 3). In the fully adjusted model, participants with both no depression and the highest levels (Q4) had the lowest risk mortality (HR, 0.32; 95% CI, 0.21–0.48) compared with participants with both depression and low levels of ALI (Q1).

**Table 3:** Joint association of advanced lung cancer inflammation index (ALI) and PHQ-9 score with all-cause mortality among chronic kidney disease patients.

Subgroup			Hazard ratio (95% CI)		
	ALI	No. of deaths (No. of participants)	Model 1^a^	Model 2^b^	Model 3^c^
PHQ-9 score ≥ 10 (depression)	Q1	45/107	1 [Reference]	1 [Reference]	1 [Reference]
	Q2	34/123	0.56 (0.36–0.86)	0.59 (0.38–0.90)	0.68 (0.45–1.04)
	Q3	25/99	0.47 (0.24–0.90)	0.66 (0.39–1.12)	0.73 (0.44–1.24)
	Q4	24/120	0.45 (0.22–0.91)	0.67 (0.33–1.39)	0.65 (0.29–1.46)
PHQ-9 score < 10 (no depression)	Q1	345/877	0.73 (0.48–1.09)	0.58 (0.39–0.86)	0.69 (0.46–1.03)
	Q2	205/860	0.41 (0.27–0.63)	0.40 (0.27–0.60)	0.50 (0.33–0.76)
	Q3	176/884	0.35 (0.23–0.53)	0.38 (0.25–0.57)	0.46 (0.29–0.72)
	Q4	131/864	0.24 (0.16–0.36)	0.27 (0.18–0.39)	0.32 (0.21–0.48)

PHQ-9, Patient Health Questionnaire-9.

a Model 1: No adjustment for covariates.

b Model 2: Adjusted for age, sex, race, PIR, education level, and marital status.

c Model 3: Adjusted for smoking, alcohol use, sleep duration, AST, ALT, HDL-C, total cholesterol, eGFR, hypertension, hyperlipidemia, CVD, DM, and cancer based on Model 2.

### 3.5 Subgroup and sensitivity analyses

In the subgroup analyses, we performed the main analyses stratified by age (<65 vs. ≥65 years), PIR (<1.3 vs. 1.3 to <3.5 vs. ≥3.5) and DM (yes vs. pre-DM vs. no). The joint effect was consistent in all subgroups, and no significant interaction was found (Supplementary Tables S2–S5).

Two sensitivity analyses were then performed to show that the combined effect remained robust. First, deaths with less than 1 year of follow-up were excluded (Supplementary Table S6). Second, no weighting was applied to the analyses (Supplementary Table S7). However, the joint effect remained robust.

## 4 Discussion

This study investigated the impact of depression and nutritional-inflammatory status (ALI) on survival in a CKD population using a prospective nationally representative cohort. First, a comparison of the ALI with the 14 indicators of nutritional/inflammation (based on blood counts) demonstrated its superior performance in predicting mortality. Second, we found that PHQ-9 score and ALI were independently associated with the risk of all-cause mortality. In addition, our findings suggest that patients with both depression and low levels of ALI (Q1) have the highest risk relative to the rest of the population. Finally, this joint effect was consistent in all subgroups, and this main association also proved robust in sensitivity analyses. This is the first study to investigate the combined impact of nutritional-inflammatory status and depression on the risk of death in patients with CKD.

Several studies have demonstrated that depressive symptoms in CKD patients are inextricably linked to the immune-inflammatory state (8–10, 35–39). These studies all concluded that depression in CKD patients tends to be associated with malnutrition and higher levels of inflammation. One of the most basic comprehensive management and dietary treatments for the CKD population is a low-protein diet (LPD) (40). Although this management approach slows the progression of CKD, it cannot be ignored that it increases the risk of malnutrition (41, 42). LPD is usually accompanied by high carbohydrate intake and acute muscle loss leading to increased levels of inflammation and insulin resistance (43–45). In addition, patients have decreased levels of certain amino acids (e.g., tyrosine and tryptophan) and micronutrients (e.g., vitamin D and zinc) (44, 45). All of these will lead to an increased risk of depression (45–47). Chronic inflammation is often present in people with CKD, and this is an important route to depression (26, 48). For example, inflammation can contribute to depression by affecting the activation of the hypothalamic-pituitary-adrenal axis and disrupting the two-way communication system between the gut microbiota and the brain (49–51).

Interestingly, depression can exacerbate levels of malnutrition and inflammation leading to a vicious cycle of disease. When depression occurs in CKD patients it can lead to loss of appetite and reduced dietary compliance, which can cause malnutrition in the organism (52, 53). It is also an indisputable fact that depression leads to increased peripheral inflammation through various mechanisms (54). For example, patients with depression have an increased release of adrenocorticotropic hormones from the central system leading to excessive activation of the hypothalamic-pituitary-adrenal axis ultimately exacerbating systemic inflammation (19). In short, depressive symptoms, malnutrition, and inflammation interact and influence each other. Nevertheless, only two studies to date have explored the combined effects of inflammation/malnutrition and depression on mortality risk (15, 55). The study populations for these two cohorts were derived from representative samples of ≥50-year-old community residents in England and ≥40-year-old cancer survivors in the US, respectively. They confirmed that the combined effects were associated with a significantly increased risk of all-cause and CVD death in men and a significantly increased risk of all-cause and non-cancer mortality in cancer survivors. Notably, similar studies have not been explored in CKD.

Most studies have shown that depression not only increases the incidence of CKD but also accelerates the progression of CKD leading to a higher risk of death (19, 56–58). Although our findings are consistent with this, this conclusion is also somewhat controversial. A recent Mendelian randomization study found that depressive symptoms are causally associated with decreased eGFR and are an important causative factor in impairing renal function, however, it is not correlated with the risk of incident of CKD (59). Furthermore, a cohort study of Chinese peritoneal dialysis patients concluded that depression was not an independent prognostic indicator of their predicted one-year mortality rate (60). Interestingly, Saglimbene et al. confirmed that depressive symptoms were only associated with the risk of non-CVD death in CKD patients on hemodialysis, but not with the risk of CVD death (61). These differences may be due to the short follow-up period, small sample size, and differences in the characteristics of the population. Moreover, depression has been associated with several comorbidities in CKD, for example, it is associated with infection in dialysis patients (62), low bone mineral density in elderly non-dialysis patients (63), and sarcopenia in patients with different stages of CKD (64). The exact mechanism by which depression are associated with a poor prognosis in CKD is unclear. According to previous studies (19, 56), this may be related to behavioral and biological factors in depressed patients. Depressed patients are more prone to many adverse health behaviors, such as obesity, decreased treatment adherence, and lack of physical activity, which are risk factors for CKD prognosis (19, 65). Depression is commonly associated with higher levels of low-grade systemic inflammation, endothelial dysfunction, abnormal activation of the hypothalamic-pituitary-adrenal axis, and overactivation of the sympathetic nervous system, which persistently interferes with the intrarenal microcirculation and perfusion distribution, leading to further renal damage in patients with CKD (66, 67).

There is a wide variety of nutritional/inflammatory markers and most studies have only explored their relationship with CKD prognosis separately (6, 11–13, 68). However, no study has compared their predictive performance in a comprehensive manner to select the best predictor. We included as many previous nutritional/inflammatory indicators as possible in this study and subsequently confirmed that ALI had the strongest predictive performance by time-ROC. On the other hand, this is the first study to investigate the association between ALI and prognosis in the CKD population. ALI was first developed to assess the prognosis of lung cancer patients and was found to be the best inflammatory biomarker of overall survival in lung cancer patients (14). Subsequently, a growing number of studies have found it to have superior predictive performance in multiple types of cancer, which include gastric, colorectal, head and neck squamous cell carcinoma, melanoma, thymic epithelial tumors, hepatocellular carcinoma, nasopharyngeal carcinoma, and diffuse large B-cell lymphoma (15, 69, 70). Interestingly, in recent years ALI has been found to have excellent predictive performance in certain non-tumor diseases as well, which include stroke, type 2 diabetes, heart failure, and hypertension (71–74). Alarmingly in this study, ALI was found to be the strongest predictor of all-cause mortality in CKD patients relative to other nutritional/inflammatory markers. This is mainly since low ALI reflects a combination of malnutrition and high levels of inflammation, which are important factors in the poor prognosis of CKD.

ALI was calculated from BMI, serum albumin level, and NLR. This is even though higher BMI is a risk factor for developing CKD (75–77). Interestingly, higher BMI was associated with a decreased risk of death in the CKD population (78, 79). CKD is a chronic inflammation-related disease prone to malnutrition leading to cachexia (79). Cachexia is commonly associated with a worsening prognosis, and BMI is one of the criteria used to diagnose cachexia (79). Serum albumin levels are also a key indicator of the body’s nutritional status, and hypoalbuminemia is a key predictor of death in patients with CKD (80). Hypoalbuminemia in the CKD population is commonly caused by chronic inflammation, dialysis treatment, catabolic alterations, and protein restriction. In addition, albumin is one of the most important antioxidants in the blood (81), and its decline exacerbates the systemic inflammatory response in CKD. Notably, when combined with the degree of systemic inflammation serum albumin can better predict the risk of death in CKD patients (82). This partly explains the superior performance of ALI predictions. NLR, derived from neutrophil and lymphocyte counts, is an important indicator of systemic inflammation and is strongly associated with the prognosis of CKD patients (83, 84). Neutrophils can cause renal fibrosis directly or indirectly by releasing reactive oxygen species, granular material, inflammatory mediators, collagen1, and pro-fibrotic inflammatory cytokines, leading to CKD progression (85). Whereas many types of lymphocytes attenuate inflammation and fibrosis in the kidney, such as IL-33R+ and IL-2Ra + regulatory T cells, INF-γ-producing CD8^+^ T cells, and CD11c^+^ CD8^+^ T cells in obstructed kidney (86, 87). In overview, it is significant to explore the combined effects of depression and ALI on the prognosis of patients with CKD.

The major strength of our study is that it used a representative sample of CKD patients in the US, as well as a multiethnic sample so that the findings can be generalized to the larger CKD population. Furthermore, reliable and exhaustive data were included in NHNAES so that we could control as much as possible for well-known confounders such as demographic characteristics, lifestyle, and history of chronic disease. We also performed some sensitivity analyses at the end to assess the robustness of the findings.

Nevertheless, some limitations must be taken into account when explaining our findings. Firstly, indicators of depression and nutritional-inflammatory status in the CKD population were measured only at the time of baseline data collection; therefore, we were unable to obtain the dynamics of these conditions during the follow-up period. Secondly, although as much information on confounders as possible was obtained, there was still residual confounding information that could not be obtained, such as medication adherence, the cycle and duration of dialysis treatments, renal transplant history, and medication use. For example, decreased medication adherence is common in depressed patients and this can have a serious impact on the prognosis of CKD patients. One study found that depression in hemodialysis patients was significantly associated with blood pressure medication nonadherence (88). This has led to confusion about whether depression itself has an impact on CKD prognosis. In addition, a portion of depressed patients may be receiving sertraline. Of note, one study found that sertraline use in CKD patients was associated with downregulation of certain otherwise high inflammatory biomarkers (89). So the inflammatory markers we measured may have been affected by these drugs as well. Therefore, the absence of above information may lead to biased study results. Thirdly, CKD populations with major depressive symptoms may disproportionately choose not to participate in the NHANES survey (90); therefore, a small proportion of major depressive populations may be absent in this study.

## 5 Conclusion

In this cohort study of CKD patients in the US, findings showed that both depression and nutritional-inflammatory status were associated with their all-cause mortality. Notably, patients with poorer nutritional-inflammatory status and depression had the worst prognosis relative to the other populations. These results emphasize that special attention should be paid to depression and nutrient-inflammatory status when developing individualized intervention strategies to improve the prognosis of patients with CKD and that they should be targeted for treatment and prevention.

## Data Availability

Publicly available datasets were analyzed in this study. This data can be found here: Publicly available datasets were analyzed in this study. This data can be found here: NHANES database (https://www.cdc.gov/nchs/nhanes/index.htm).

## Footnotes

^1^ https://ftp.cdc.gov/pub/Health_Statistics/NCHS/datalinkage/linked_mortality/
