# Supplementary material for: Independent and joint influence of depression and advanced lung cancer inflammation index on mortality among individuals with chronic kidney disease
Source: Front Nutr. 2024 Oct 23;11:1453062. doi: 10.3389/fnut.2024.1453062 (PMC11539836; doi:10.3389/fnut.2024.1453062)
Supplement: Supplementary file 1 [file Table_1.DOCX]

Supplementary Material

# Independent and joint influence of depression and advanced lung cancer inflammation index on mortality among individuals with chronic kidney disease

**Jie Zhou^1,2†^, Wenjun Liu^3†^, Xiaoxin Liu^4†^, Jijun Wu^5, 6^, Ying Chen^1^***

^1^NHC Key Laboratory of Hormones and Development, Tianjin Key Laboratory of Metabolic Diseases, Chu Hsien-I Memorial Hospital & Tianjin Institute of Endocrinology, Tianjin Medical University, Tianjin 300134, China;

^2^Department of Gastrointestinal Oncology Surgery, Hubei Cancer Hospital, Tongji Medical College, Huazhong University of Science and Technology, Wuhan 430079, Hubei, China;

^3^Department of Vascular Surgery, Guangdong Provincial Key Laboratory of Major Obstetric Diseases, Guangdong Provincial Clinical Research Center for Obstetrics and Gynecology, The Third Affiliated Hospital, Guangzhou Medical University, Guangzhou 510150, China;

^4^Department of Nephrology, Liyuan Hospital, Tongji Medical College, Huazhong University of Science and Technology, Wuhan 430077, Hubei, China;

^5^Department of interventional radiology, Zhongshan Torch Development Zone People's Hospital, Zhongshan, Guangdong 528436, China;

^6^Third Clinical School, Guangzhou Medical University, Guangzhou 510150, China.

*** Correspondence:**

**Ying Chen**

**E-mail: ying.chen@tmu.edu.cn**

**^†^ These authors contributed equally to this work and share first authorship**

Table S1. Calculation of various nutritional/inflammatory indicators

| **Indicators** | **Definition or calculation formula** |
| --- | --- |
| **ALI** | BMI (kg/m^2^) × albumin (g/dl) / NLR |
| **NLR** | neutrophil count (×10^9^) / lymphocyte count (×10^9^) |
| **SII** | platelet count (×10^9^) × neutrophil count (×10^9^) / lymphocyte count (×10^9^) |
| **SIRI** | neutrophil count (×10^9^) × monocyte count (×10^9^) / lymphocyte count (×10^9^) |
| **PIV** | neutrophil count (×10^9^) × platelet count (×10^9^) × monocyte count (×10^9^) /lymphocyte count (×10^9^) |
| **PLR** | platelet count (×10^9^)/lymphocyte count (×10^9^) |
| **PNI** | albumin (g/L) + 5×lymphocyte count (×10^9^) |
| **GNRI** | 1.489×albumin (g/L) + (41.7× current weight/ ideal body weight (IBW) |
| **PINI** | albumin (g/mL) × 0.9 - monocyte count (/mm^3^) × 0.0007 |
| **NHR** | neutrophil count (×10^3^ cells/ul) / high-density lipoprotein cholesterol (mg/dL) |
| **MHR** | monocyte count (×10^3^ cells/ul) / high-density lipoprotein cholesterol (mg/dL) |
| **PHR** | platelet count (×10^3^ cells/ul) / high-density lipoprotein cholesterol (mg/dL) |
| **LHR** | lymphocyte count (×10^3^ cells/ul) / high-density lipoprotein cholesterol (mg/dL) |
| **CONUT score** |  |
| Serum albumin (g/L) | albumin score |
| ≥ 35 | 0 |
| 30 to < 35 | 2 |
| 25 to < 30 | 4 |
| < 25 | 6 |
| Total lymphocytes (×10^9^/L) | Total-lymphocyte score |
| > 1.6 | 0 |
| 1.2 to 1.6 | 1 |
| 0.8 to <1.2 | 2 |
| < 0.8 | 3 |
| Total cholesterol (mg/dL) | Total-cholesterol score |
| > 180 | 0 |
| 140-180 | 1 |
| 100 to < 140 | 2 |
| < 100 | 3 |
| CONUT score (total) | albumin score+ TLC score+ Total-cholesterol score |

Abbreviation: ALI, advanced lung cancer inflammation index; NLR, neutrophil to lymphocyte ratio; SII, systemic immune-inflammation index; SIRI, systemic inflammation response index; PIV, pan-immune-inflammation value; PLR, platelet-to-lymphocyte ratio; PNI, prognostic nutritional index; GNRI, geriatric nutritional risk index; PINI, prognostic immune nutritional index; NHR, neutrophil to high-density lipoprotein-cholesterol ratio; MHR, Monocyte to high-density lipoprotein-cholesterol ratio; PHR, platelet to high-density lipoprotein-cholesterol ratio; LHR, lymphocyte to high-density lipoprotein-cholesterol ratio; COUNT score, controlling nutritional status score.

IBW for males and females were calculated using the formulas height (cm)-100 - [height (cm)-150/4] and height (cm)-100 - [height (cm)-150/2.5], respectively.

Table S2. Joint association of advanced lung cancer inflammation index (ALI) and PHQ-9 score with all-cause mortality among CKD patients stratified by age

| **Subgroup** |  | < 65 years | | ≥ 65 years | |
| --- | --- | --- | --- | --- | --- |
|  |  | Hazard ratio (95% CI) | *p* value | Hazard ratio (95% CI) | *p* value |
| PHQ-9 score≥ 10 | Low ALI | 1 [Reference] |  | 1 [Reference] |  |
|  | High ALI | 0.52 (0.29-0.92) | 0.024 | 0.87 (0.54-1.38) | 0.547 |
| PHQ-9 score< 10 | Low ALI | 0.71 (0.43-1.17) | 0.176 | 1.01 (0.73-1.41) | 0.929 |
|  | High ALI | 0.38 (0.23-0.63) | <0.001 | 0.62 (0.44-0.86) | 0.004 |
| *p* for interaction | 0.226 | | | | |

Abbreviation: PHQ-9, Patient Health Questionnaire-9; CKD, chronic kidney disease;

Adjusted for sex, race, PIR, education level, marital status, smoking, alcohol use, sleep duration, AST, ALT, HDL-C, total cholesterol, eGFR, hypertension, hyperlipidemia, CVD, DM, and cancer.

Table S3. Joint association of advanced lung cancer inflammation index (ALI) and PHQ-9 score with all-cause mortality among CKD patients stratified by sex

| **Subgroup** |  | Male | | Female | |
| --- | --- | --- | --- | --- | --- |
|  |  | Hazard ratio (95% CI) | *p* value | Hazard ratio (95% CI) | *p* value |
| PHQ-9 score≥ 10 | Low ALI | 1 [Reference] |  | 1 [Reference] |  |
|  | High ALI | 0.66 (0.38-1.16) | 0.150 | 0.87 (053-1.43) | 0.590 |
| PHQ-9 score< 10 | Low ALI | 0.81 (0.57-1.16) | 0.254 | 0.84 (0.55-1.26) | 0.393 |
|  | High ALI | 0.45 (0.31-0.65) | <0.001 | 0.54 (0.36-0.82) | 0.003 |
| *p* for interaction | 0.717 | | | | |

Abbreviation: PHQ-9, Patient Health Questionnaire-9; CKD, chronic kidney disease;

Adjusted for age, race, PIR, education level, marital status, smoking, alcohol use, sleep duration, AST, ALT, HDL-C, total cholesterol, eGFR, hypertension, hyperlipidemia, CVD, DM, and cancer.

Table S4. Joint association of advanced lung cancer inflammation index (ALI) and PHQ-9 score with all-cause mortality among CKD patients stratified by diabetes

| Subgroup |  | Diabetes | | Pre-diabetes | | Non-diabetes | |
| --- | --- | --- | --- | --- | --- | --- | --- |
|  |  | Hazard ratio (95% CI) | *p* value | Hazard ratio (95% CI) | *p* value | Hazard ratio (95% CI) | *p* value |
| PHQ-9 score≥ 10 | Low ALI | 1 [Reference] |  | 1 [Reference] |  | 1 [Reference] |  |
|  | High ALI | 0.77 (0.48-1.23) | 0.275 | 0.20 (0.05-0.80) | 0.024 | 0.89 (0.48-1.66) | 0.722 |
| PHQ-9 score< 10 | Low ALI | 1.07 (0.75-1.52) | 0.713 | 0.12 (0.04-0.34) | <0.001 | 0.75 (0.47-1.19) | 0.220 |
|  | High ALI | 0.60 (0.42-0.86) | 0.005 | 0.10 (0.04-0.27) | <0.001 | 0.46 (0.28-0.74) | 0.002 |
| p for interaction | 0.593 | | | | | | |

Abbreviation: PHQ-9, Patient Health Questionnaire-9; CKD, chronic kidney disease;

Adjusted for age, sex, race, PIR, education level, marital status, smoking, alcohol use, sleep duration, AST, ALT, HDL-C, total cholesterol, eGFR, hypertension, hyperlipidemia, CVD, and cancer.

Table S5. Joint association of advanced lung cancer inflammation index (ALI) and PHQ-9 score with all-cause mortality among CKD patients stratified by PIR

| Subgroup |  | PIR< 1.3 | | 1.3≤ PIR< 3.5 | | PIR≥ 3.5 | |
| --- | --- | --- | --- | --- | --- | --- | --- |
|  |  | Hazard ratio (95% CI) | *p* value | Hazard ratio (95% CI) | *p* value | Hazard ratio (95% CI) | *p* value |
| PHQ-9 score≥ 10 | Low ALI | 1 [Reference] |  | 1 [Reference] |  | 1 [Reference] |  |
|  | High ALI | 0.87 (0.53-1.41) | 0.563 | 0.56 (0.30-1.05) | 0.068 | 0.78 (0.22-2.78) | 0.701 |
| PHQ-9 score< 10 | Low ALI | 1.02 (0.69-1.51) | 0.913 | 0.66 (0.43-1.02) | 0.061 | 0.35 (0.17-0.76) | 0.007 |
|  | High ALI | 0.55 (0.37-0.82) | 0.003 | 0.43 (0.28-0.67) | <0.001 | 0.26 (0.12-0.56) | <0.001 |
| p for interaction | 0.345 | | | | | | |

Abbreviation: PHQ-9, Patient Health Questionnaire-9; CKD, chronic kidney disease; PIR, income-poverty ratio

Adjusted for age, sex, race, education level, marital status, smoking, alcohol use, sleep duration, AST, ALT, HDL-C, total cholesterol, eGFR, hypertension, hyperlipidemia, CVD, DM, and cancer.

Table S6. Joint association of advanced lung cancer inflammation index (ALI) and PHQ-9 score with all-cause mortality after exclusion of deaths within the first 1 year of follow-up

|  |  | **Hazard ratio (95% CI)** | | |
| --- | --- | --- | --- | --- |
| **Subgroup** | ALI | Model 1^a^ | Model 2^b^ | Model 3^c^ |
| PHQ-9 score≥ 10  (Depression) | Q1 | 1 [Reference] | 1 [Reference] | 1 [Reference] |
|  | Q2 | 0.67 (0.43-1.05) | 0.69 (0.44-1.07) | 0.81 (0.53-1.25) |
|  | Q3 | 0.56 (0.29-1.09) | 0.80 (0.47-1.34) | 0.86 (0.51-1.44) |
|  | Q4 | 0.45 (0.21-0.97) | 0.65 (0.30-1.43) | 0.62 (0.27-1.44) |
| PHQ-9 score< 10  (No depression) | Q1 | 0.83 (0.55-1.26) | 0.63 (0.43-0.93) | 0.74 (0.50-1.10) |
|  | Q2 | 0.49 (0.31-0.77) | 0.46 (0.30-0.69) | 0.57 (0.37-0.87) |
|  | Q3 | 0.42 (0.28-0.65) | 0.44 (0.30-0.67) | 0.53 (0.34-0.83) |
|  | Q4 | 0.29 (0.19-0.46) | 0.31 (0.21-0.47) | 0.37 (0.24-0.57) |

Abbreviation: PHQ-9, Patient Health Questionnaire-9;

^a^ Model 1: No adjustment for covariates;

^b^ Model 2: Adjusted for age, sex, race, PIR, education level, and marital status;

^c^ Model 3: Adjusted for smoking, alcohol use, sleep duration, AST, ALT, HDL-C, total cholesterol, eGFR, hypertension, hyperlipidemia, CVD, DM, and cancer based on Model 2.

Table S7. Joint association of advanced lung cancer inflammation index (ALI) and PHQ-9 score with all-cause mortality among CKD patients (unweighted analyses)

|  |  | **Hazard ratio (95% CI)** | | |
| --- | --- | --- | --- | --- |
| **Subgroup** | ALI | Model 1 | Model 2 | Model 3 |
| PHQ-9 score≥ 10  (Depression) | Q1 | 1 [Reference] | 1 [Reference] | 1 [Reference] |
|  | Q2 | 0.54 (0.34-0.84) | 0.60 (0.39-0.94) | 0.70 (0.44-1.09) |
|  | Q3 | 0.51 (0.31-0.84) | 0.76 (0.47-1.25) | 0.83 (0.51-1.37) |
|  | Q4 | 0.38 (0.23-0.63) | 0.63 (0.38-1.04) | 0.65 (0.39-1.08) |
| PHQ-9 score< 10  (No depression) | Q1 | 0.89 (0.65-1.21) | 0.68 (0.50-0.94) | 0.81 (0.59-1.12) |
|  | Q2 | 0.48 (0.35-0.66) | 0.46 (0.33-0.64) | 0.57 (0.41-0.80) |
|  | Q3 | 0.38 (0.28-0.53) | 0.40 (0.29-0.56) | 0.48 (0.35-0.68) |
|  | Q4 | 0.28 (0.20-0.40) | 0.31 (0.22-0.44) | 0.39 (0.27-0.55) |

Abbreviation: PHQ-9, Patient Health Questionnaire-9;

^a^ Model 1: No adjustment for covariates;

^b^ Model 2: Adjusted for age, sex, race, PIR, education level, and marital status;

^c^ Model 3: Adjusted for smoking, alcohol use, sleep duration, AST, ALT, HDL-C, total cholesterol, eGFR, hypertension, hyperlipidemia, CVD, DM, and cancer based on Model 2.
